# 新型冠状病毒肺炎疫情防控常态化阶段的异基因造血干细胞移植

**DOI:** 10.3760/cma.j.cn121090-20240817-00308

**Published:** 2024-11

**Authors:** 启发 刘, 韧 林

**Affiliations:** 南方医科大学南方医院血液科，南方医科大学血液病研究所，广东省血液系统疾病临床医学研究中心，广州 510515 Department of Hematology, Nanfang Hospital, Southern Medical University, Clinical Medical Research Center of Hematological Diseases of Guangdong Province, Guangzhou 510515, China

## Abstract

目前全球已经进入新型冠状病毒肺炎（COVID-19）疫情防控常态化阶段。COVID-19仍将在较长时间内影响异基因造血干细胞移植（allo-HSCT）患者，作者探讨现阶段COVID-19对allo-HSCT策略及预后的可能影响。移植前感染SARS-CoV-2的患者移植时机选择在症状缓解且SARS-CoV-2转阴后为优。供者采集时感染或并不影响采集。allo-HSCT患者移植早期发生COVID-19仍有较高的重症发生率；重症COVID-19仍然是移植后非复发死亡和生存的危险因素。COVID-19与移植后并发症如其他感染、内皮损伤相关并发症及复发的关系有待进一步大样本研究。

## Abstract (en)

At present, the world has entered the normalization stage of coronavirus disease 2019（COVID-19）management. COVID-19 continues to affect patients with allogeneic hematopoietic stem cell transplantation（allo-HSCT）for a long period. The author discussed the possible effect of COVID-19 on HSCT strategy and prognosis during this period based on literature reports. Transplantation should be deferred until clinical resolution and negative severe acute respiratory syndrome coronavirus 2（SARS-CoV-2）in patients infected with SARS-CoV-2 before transplantation. Donors infected with SARS-CoV-2 during the stem cell collection may not affect apheresis. Allo-HSCT recipients demonstrated a high rate of severe COVID-19 if COVID-19 occurred at the early stage of transplantation. Severe COVID-19 remains a risk factor for nonrelapse mortality and survival after transplantation. The association between COVID-19 and post-transplantation complications, such as other infections, endothelial injury-related complications, and relapse, needs to be further investigated in large samples.

新型冠状病毒肺炎（COVID-19）是由SARS-CoV-2冠状病毒引起的疾病。根据世界卫生组织（WHO）公布的数据，全球COVID-19病例已超过7亿，死亡病例超过700万例。尽管WHO于2023年5月宣布COVID-19的紧急阶段已经结束，SARS-CoV-2仍然在社区及医院持续存在并传播，且在短期之内COVID-19不会消失。COVID-19对造血干细胞移植（HSCT）尤其是异基因造血干细胞移植（allo-HSCT）的影响从疫情初期即得到广泛关注，发展为中重型COVID-19的allo-HSCT患者预后极差。SARS-CoV-2从原始病毒株开始已经历十余个变异株，奥密克戎变异株自2021年11月被发现以来目前仍然是全球主要流行株。随着毒株的变化及疫苗的应用等，COVID-19对HSCT患者生存的影响似乎在逐渐下降，但对HSCT相关并发症的影响仍并不十分清楚。本文结合国内外文献报道，探讨COVID-19疫情防控常态化阶段下的移植策略及COVID-19对预后的可能影响。

一、COVID-19患者移植时机

移植前感染SARS-CoV-2的患者何时可行HSCT临床上仍在探讨。美国移植与细胞治疗协会（ASTCT）发布的指南建议对于无症状的SARS-CoV-2感染者推迟14 d行HSCT，有症状者需待症状缓解且自新冠检测阳性起至少推迟14～20 d。ASTCT指南认为因为存在长时间的病毒排出（prolonged shedding），移植前核酸转阴并非必需[1]。欧洲白血病感染会议（ECIL）及欧洲血液及骨髓移植协会（EBMT）推荐有症状感染者需待症状缓解且SARS-CoV-2转阴后为优；如果PCR检测持续阳性但急需移植，建议行抗病毒治疗，疗程根据移植计划决定[2]。长时间病毒排出在血液病患者中并不鲜见，因此这部分患者是否可行移植是临床上关注的重点。目前核酸阳性情况下行HSCT仅见个案报道。Krajewski等[3]报道了2例急需移植的难治复发血液病患儿在无症状且SARS-CoV-2持续阳性情况下行allo-HSCT，Oltolin等[4]亦报道了1例持续SARS-CoV-2阳性的成人患者行HSCT。移植后3例患者均未出现COVID-19复燃，其中1例因多器官功能衰竭在+13 d死亡，2例无病生存。笔者认为对于移植时机的选择仍应优选核酸转阴后，对于急需移植的患者在积极抗病毒治疗情况下需充分评估移植风险及获益。

二、COVID-19阳性供者

在COVID-19大流行时期，考虑到供者感染风险、交通以及移植物病毒传播风险等问题，多个国际指南推荐提前冻存供者造血干细胞。在新冠常态化阶段，供者SARS-CoV-2阳性移植不可避免，提前冻存策略很难符合临床实际需求。在2022年12月国内防疫政策调整后，各移植中心均报道了供者在采集前或采集当日SARS-CoV-2阳性病例[5]–[6]。此前国外已有数例供者SARS-CoV-2阳性的个案报道，采集物中SARS-CoV-2核酸检均为阴性，采集未受影响[7]–[9]。国内苏州大学附属第一医院和北京大学人民医院分别报道了12例和16例供者SARS-CoV-2阳性移植，对比同期供者SARS-CoV-2阴性移植，植入率差异均无统计学意义[5]–[6]。南方医科大学南方医院等开展的多中心研究对比了9例供者SARS-CoV-2阳性、45例供者SARS-CoV-2既往感染和20例供者未感染过SARS-CoV-2的移植预后，三组采集物淋巴细胞亚群、植入和生存差异均无统计学意义，移植物SARS-CoV-2检测均为阴性，值得注意的是供者SARS-CoV-2阳性组Ⅱ～Ⅳ度急性移植物抗宿主病（aGVHD）发生率高于其他两组[10]。北京大学人民医院近期发表的一项研究也显示采集时供者SARS-CoV-2阳性或供者处于转阴30 d内是Ⅱ～Ⅳ度和Ⅲ～Ⅳ度aGVHD的危险因素[11]。引起这一现象的原因尚不清楚，SARS-CoV-2是否影响采集物某些特殊细胞亚群或是细胞因子的变化从而导致GVHD发生率升高，值得进一步研究。因此，笔者认为供者阳性可能不影响采集及造血重建，仍应警惕其对GVHD的影响。

三、移植患者COVID-19的临床特征与转归

HSCT患者COVID-19的感染率各家报道不一。一项荟萃分析显示allo-HSCT患者的COVID-19感染率为93％（全部纳入研究均为2020年发表）[12]。在我国防疫策略调整后，中国医学科学院血液病医院的研究显示allo-HSCT患者COVID-19感染率为67.0％[13]，而南方医科大学南方医院的数据显示感染率为79.57％。HSCT患者COVID-19症状个体差异大，受到免疫状态、合并症等因素影响。相较于原始株和delta株，目前仍作为主要流行株的奥密克戎及其变异株引起的感染临床症状较轻。在奥密克戎株流行时期，HSCT患者与健康人群COVID-19临床症状是否存在差异鲜有研究。笔者团队总结了2022年12月后南方医科大学南方医院感染SARS-CoV-2的allo-HSCT患者331例，对比患者密接的817例SARS-CoV-2感染免疫健全者，发现HSCT患者无症状的比例相对较低（0.91％对4.28％，*P*＝0.009），但发热及高热（>39 °C）的比例低于健康人群（发热：65.86％对74.17％，*P*＝0.005；高热：31.42％对54.10％，*P*<0.001），同时上呼吸道症状的比例亦低于健康人群（25.98％对69.77％，*P*<0.001）；但allo-HSCT患者移植后100 d内发生中重型COVID-19的比例达到34.14％，而移植后100 d至1年及移植后1年至2年的中重型比例分别为18.90％和3.68％。相对于健康人群极低的中重型比例，文献报道仍有15％左右的allo-HSCT患者会发展为COVID-19[14]–[15]。国内研究显示在奥密克戎株流行时期，长期应用免疫抑制剂以及移植早期阳性是发展为中重型COVID-19的主要危险因素[15]–[16]。文献报道HSCT患者COVID-19临床缓解中位时间在2～4周，但部分患者存在持续病毒排出，可达200 d以上[15]–[18]。临床上经过多疗程及多种抗病毒药物治疗核酸持续阳性的情况亦不少见，且部分患者仍有肺部感染相关症状。因此，对于HSCT患者SARS-CoV-2感染，尤其是移植早期感染仍需重视。

四、COVID-19对移植后其他感染的影响

重症COVID-19合并其他病原体感染已有大量报道[19]–[22]，但聚焦HSCT患者COVID-19感染时合并其他病原菌感染情况以及既往COVID-19感染史是否影响其他病原感染的易感性报道较少。一项包含193例肺移植患者的美国单中心回顾性研究显示在取消严格COVID-19防疫措施后，肺移植患者外周血及肺泡灌洗液细菌的检出率均增高。这一结果提示COVID-19对免疫低下患者细菌易感性可能存在影响[23]。一项来自俄罗斯的研究对比了55例移植前有SARS-CoV-2感染史的HSCT受者和122例未感染SARS-CoV-2的HSCT受者，结果显示2年累积血流感染发生率和细菌谱差异均无统计学意义[24]。在真菌感染方面，免疫低下人群包括血液肿瘤和HSCT患者是COVID-19合并肺曲霉病、毛霉病的高危人群[19]–[20]。Siniaev等[24]的研究显示移植前有SARS-CoV-2感染史的HSCT受者移植后2年累积侵袭性真菌病发生率为21.5％，而无感染史的HSCT受者为10.5％（*P*＝0.08）。自疫情初期，COVID-19重症患者合并EB病毒（EBV）感染即被高度关注[25]，荟萃分析显示COVID-19患者活动性EBV感染发生率高于非COVID-19患者[21]。引起这一现象的机制尚不清楚，一些观点认为与COVID-19患者淋巴细胞减少、SARS-CoV-2介导机体抗病毒免疫逃逸等相关[25]–[26]。Wen等[27]报道移植患者COVID-19后150 d累积EBV血症发生率在重症和非重症患者中分别为7.4％和0。另一方面EBV被认为是引起长新冠综合征（Long COVID-19）的原因之一，可能的机制为EBV上调ACE2表达有利于SARS-CoV-2持续感染等[28]–[29]。除EBV外，COVID-19患者合并巨细胞病毒（CMV）和其他疱疹病毒亦有一些报道[30]。北京大学人民医院的回顾性研究显示重症COVID-19移植患者和长程感染HSCT患者中CMV病和CMV肺炎的发生率显著升高[27]。需要注意的是，一项较小样本的回顾性研究显示移植前有SARS-CoV-2感染史对移植后病毒感染的发生率并无影响[24]。笔者认为鉴于SARS-CoV-2对机体免疫的潜在影响，需要更多循证医学证据探讨前驱SARS-CoV-2感染史对移植患者其他感染的影响。

五、COVID-19对移植后内皮损伤相关并发症的影响

血管内皮细胞损伤可能是引起HSCT后一系列并发症的原因，目前认为其机制主要包括免疫细胞互作、补体激活和炎症因子的释放等[31]。内皮损伤相关并发症包括GVHD、肝窦隙阻塞综合征（SOS/VOD）、移植相关血栓性微血管病（TA-TMA）、毛细血管渗漏、植入综合征和体液潴留等[31]。而重症COVID-19的特征之一正是内皮细胞损伤导致的微血栓形成，因此COVID-19是否增加HSCT患者这些并发症的发生风险或加剧原有内皮损伤相关并发症的严重程度是临床上亟待探讨的问题。但目前相关报道较少，且多为小样本。Niederwieser等[32]分析了14例移植前有COVID-19病史且已康复的HSCT患者，3例患者移植后分别发生SOS、TMA和TMA合并SOS，4例发生多浆膜腔积液。另一项小样本回顾性研究对比了移植前、后发生COVID-19和未发生COVID-19的患者内皮损伤相关并发症的发生率，移植前有COVID-19病史的HSCT患者TA-TMA的发生率显著高于既往无COVID-19病史者[33]。Nimgaonkar等[34]开展的回顾性研究纳入15例移植前90 d内有COVID-19病史的患者，在移植后100 d内8例患者发生aGVHD（其中1例为Ⅲ～Ⅳ度），2例SOS和1例TA-TMA。北京大学人民医院的回顾性研究纳入了179例HSCT后COVID-19患者，结果提示COVID-19可能与急慢性GVHD风险并无相关性[27]。笔者认为因SARS-CoV-2对内皮功能的损伤，大样本研究评估COVID-19对HSCT后内皮损伤相关并发症的影响具有临床指导意义。

六、COVID-19对移植后原发病复发与生存的影响

COVID-19与HSCT后原发病复发的关系报道较少，移植后SARS-CoV-2感染是否影响复发目前尚不清楚。一项小样本研究显示移植前COVID-19病史对HSCT后原发病复发并无影响[24]。后续仍需要大样本队列研究揭示COVID-19对复发的影响。随着毒株变异、疫苗和防治手段的改进等，allo-HSCT患者COVID-19后12周生存率从疫情初期的不到80％上升至奥密克戎株流行时期的95％以上[14]。国内的研究显示allo-HSCT患者COVID-19感染后4周的生存率均在98％以上[15]–[16]。值得注意的是，重症和长程感染患者移植后150 d的非复发死亡仍高达39.5％和20.0％，重症COVID-19是移植后非复发死亡和生存的危险因素[16]。

七、COVID-19疫苗与移植

新冠病毒变异株的出现使基于原始毒株研制的疫苗保护效力下降，但接种疫苗仍具有降低重症发生率及病死率的作用。新冠疫苗接种后的免疫应答与患者的免疫功能状态密切相关，文献报道allo-HSCT 6个月后且无aGVHD的患者接种疫苗后，抗体阳性率为77.6％，慢性GVHD及无免疫抑制治疗的患者中和抗体水平较高[35]。《成人血液病患者接种新型冠状病毒疫苗中国专家共识（2023年版）》[35]建议allo-HSCT患者根据疾病状态和造血及免疫功能恢复情况，在细胞回输3～6个月后接种疫苗；存在中重度或难治性GVHD或广泛型慢性GVHD且尚在应用免疫抑制剂者暂缓接种。此外，部分疫苗具有潜在引起血细胞减少、噬血细胞综合征、自身免疫性溶血等血液学不良反应的风险，临床上需谨慎评估。

八、展望

在COVID-19疫情防控常态化阶段，COVID-19对移植的挑战可能远不止以上。SARS-CoV-2对机体影响的研究尚在不断深入，因此是否需要常规监测患者免疫细胞、内皮细胞等功能从而个体化调整预处理、GVHD预防及感染防治策略等可能是未来需要面对的问题。治疗COVID-19合并其他病原体感染及其所导致的并发症也仍然是临床上尚未满足的需求。
